# Splenic Trauma during Colonoscopy: The Role of Intra-Abdominal Adhesions

**DOI:** 10.1155/2018/4879413

**Published:** 2018-05-15

**Authors:** Chukwunonso Chime, Charbel Ishak, Kishore Kumar, Venkata Kella, Sridhar Chilimuri

**Affiliations:** Bronx Care Hospital Health System, Affiliate of Icahn School of Medicine at Mount Sinai, New York, NY, USA

## Abstract

Splenic rupture following colonoscopy is rare, first reported in 1974, with incidence of 1–21/100,000. It is critical to anticipate splenic trauma during colonoscopy as one of the causes of abdominal pain after colonoscopy especially when located in the left upper quadrant or left shoulder. Postoperative adhesions is a predisposing factor for splenic injury, and management is either operative or nonoperative, based on hemodynamic stability and/or extravasation which can be seen on contrast-enhanced CT scan of the abdomen. We present a case of a splenic rupture after colonoscopy in a patient with splenocolic adhesions, requiring splenectomy as definite treatment.

## 1. Introduction

Colonoscopy is a reliable and widely used procedure for the diagnosis and management of colorectal disorders, but it is not without its risks, with the commonest being bleeding and perforation, with estimated incidences of 1.8–2.5% and 0.34–2.14%, respectively [1]. Splenic rupture is a very rare complication following colonoscopy, with 103 cases published until the end of 2012 [1]. It has an incidence of 1–21/100,000 with the first case reported in 1974 [2]. These patients are mainly female (71.5%) and have a mean age of 63 years as well as a previous history of abdominal surgery (50.8–65%) [1]. It is essential to identify this complication and to treat it as soon as possible, due to the elevated morbidity and mortality [3, 4]. We present a case of a splenic rupture after screening colonoscopy resulting in splenectomy.

## 2. Case Report

**Figure 1:**
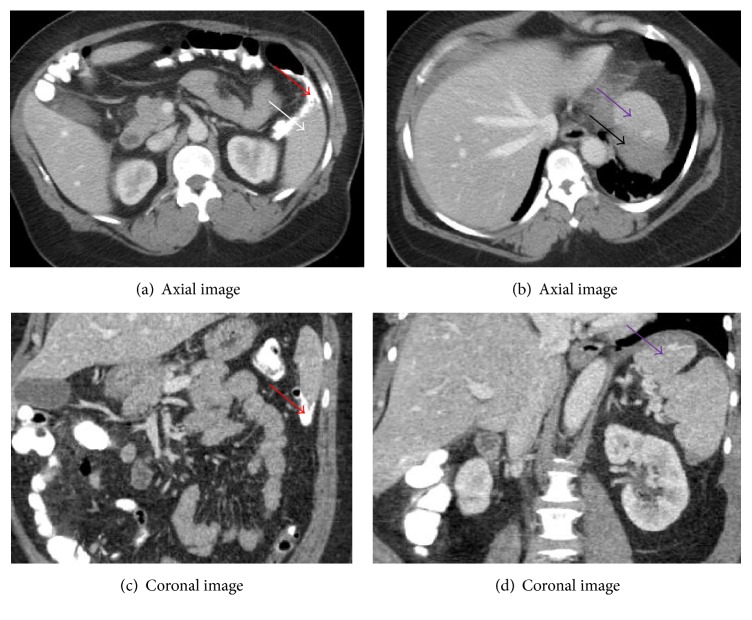
CT, 05/05/17: perisplenic and subcapsular hematoma (black arrow) along the dome of the spleen, with an area of active bleeding along the superior subcapsular region (purple arrows) with no clear fat plane separation between the colonic splenic flexure and the lower portion of the spleen (white arrows) and contrast tracking irregularly along diverticula/splenocolic adhesions and splenocolic ligament (red arrows).

51-year-old female presented to our emergency room with abdominal pain, one day after a routine screening colonoscopy done in an outside facility. Patient had colonoscopy done in the morning of the previous day and by evening she noticed a sharp left upper quadrant abdominal pain with radiation to the left shoulder, which is aggravated by respiration. Her medical history is remarkable for hypertension, diabetes, and obstructive sleep apnea. She had colonoscopy in 2009 for iron deficiency anemia and hysterectomy done in 2010. In the emergency room, she had a blood pressure of 124/67 mmhg and pulse rate of 75 bpm; respiratory rate was within normal limits. Her laboratory results revealed hemoglobin of 11.9 g/dl, white cell count of 11.1 k/ul ml, platelets of 221 k/ul ml, INR of 1.0, BUN of 14 mg/dl, and creatinine of 0.7 mg/dl. Physical examination revealed no abdominal distension, but tenderness of the left upper and lower quadrants, guarding but no rebound. Chest radiograph did not reveal any pneumothorax or air under diaphragm. Computerized tomography (CT) of the abdomen revealed a 4 × 7 cm perisplenic/subcapsular hematoma with an area of active bleeding along the subcapsular region and possible splenocolic adhesions (see Figures 1(a)–1(d)). She was admitted under the care of the surgical service and managed conservatively for 4 days, during which she received 2 packed red cells for drop in hemoglobin. On day four of admission, she was deemed to have failed conservative management evidenced by continued drop in hemoglobin despite transfusion, tachycardia, and a repeat CT of the abdomen demonstrating increasing size of splenic hematoma with perisplenic, perihepatic, and pelvic hemorrhagic ascites. Patient was then taken to the operating room for laparoscopic splenectomy. On the fourth postoperative day with updated vaccination and uneventful recovery, she was discharged home.

## 3. Discussion

Colonoscopy is the procedure most related to iatrogenic splenic injury; however, it is infrequent to have splenic injury as a complication of colonoscopy [5]. Predisposing factors for splenic trauma include splenomegaly, adhesions related to previous operations, anticoagulant use, smoking, inflammatory bowel disease, difficult colonoscopy, intention to rush during the procedure, and insufficient visualization due to inadequate bowel cleansing [6]. In addition, during colonoscopy, maneuvers such as hooking the splenic flexure to straighten left colon, applying external pressure on the left hypochondrium, slide by advancement, and alpha maneuver, can be risk factors for splenic injury [7]. Left lateral position can be considered a protective factor; it reduces the excessive traction of the splenocolic ligament or possible adhesions, as the spleen and the splenic angle of the colon approximate in the left side [8].

Symptoms in most cases begin in the first 24 hours after colonoscopy [8] but may be rarely delayed and it must be kept in mind that symptoms can present after several days [9]. The first and second most common symptoms are left upper quadrant pain with prevalence of 93% and left shoulder pain in 88%, respectively; the latter is caused by diaphragmatic irritation, related to distention of splenic capsule following bleeding [6]. Similar symptoms presenting within 24 hours of colonoscopy prompted our patient for her emergency room visit.

Diagnosis can be challenging as abdominal discomfort is common after colonoscopy due to trapped air in the colon leading to misdiagnoses of some cases of mild splenic rupture [1, 8]. Although computerized tomography is highly sensitive, suspecting this complication is the best way to assist in the early diagnosis [10]. Contrast-enhanced CT scan of the abdomen is the gold standard for diagnosis as it can describe the splenic injury grading in accordance with the American Association for the Surgery of Trauma (AAST) [11]. According to Corcillo et al., ultrasound (conventional and contrast-enhanced) provides a good alternative in patients with contraindications to CT contrast agents and in hemodynamically compromised patients (focused assessment with sonography for trauma (FAST)) [12].

Therapeutic options available for treatment include conservative management, surgery, or embolization of the splenic artery [12] depending on type of injury and hemodynamic stability. In hemodynamically unstable patients with ongoing bleeding, splenectomy is a common treatment option [1, 12]. Failure rate in patients initially treated conservatively and eventually requiring a splenectomy or embolization can be up to 44% [12]. Our patient was initially managed conservatively, but she continued to bleed as evidenced by further drop in hemoglobin and imaging evidence of the same. Options of embolization versus surgery were considered and we decided that splenectomy would lead to a more favorable outcome given her instability.

Given the important immune functions of the spleen, it is imperative not to overlook the importance of vaccination against encapsulated organisms in these patients, as they are prone to overwhelming sepsis from the organisms. According to Hammerquist et al., in nonemergent cases, vaccination against encapsulated organisms should be attempted ideally 2 weeks before surgery; however, during emergency splenectomy, vaccination should be performed until 2 weeks postoperatively [13, 14].

**Figure 2:**
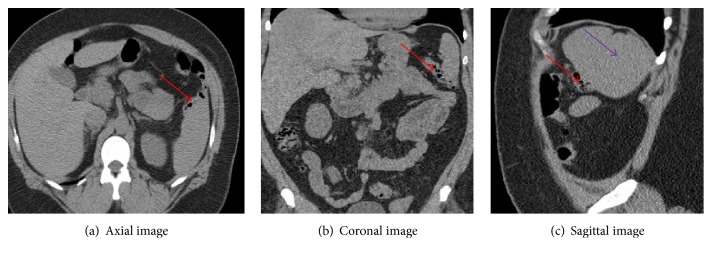
CT, 06/06/12: diverticula abutting the splenic capsule of the lower pole (red arrows) with splenomegaly (purple arrow).

We suggest that the presence of possible splenocolic adhesions, as evidenced by colonic diverticula abutting on splenic capsule, seen on abdominal imaging done before index elective colonoscopy (see Figures 2(a)–2(c)), would have contributed to her risk of splenic trauma during the colonoscopy. Laparotomy with splenectomy was decided as patient had progression of hemoperitoneum and hemodynamic instability.

## 4. Conclusion

Splenic trauma during colonoscopy is quite a rare occurrence, but anticipating it as one of the causes of abdominal pain after colonoscopy is critical especially when located in the left upper quadrant or left shoulder. Multiple risk factors have been described in literature; a few may have culminated in splenic trauma in our patient during index colonoscopy, notably splenocolic adhesions, and splenomegaly. The gold standard diagnostic examination is the contrast-enhanced CT scan of the abdomen; other diagnostic options include ultrasound (conventional and contrast-enhanced). Management of splenic injury may be one of 3 therapeutic options: conservative, surgery, or embolization of the splenic artery, based on patient's hemodynamics and ongoing extravasation.
